# Association of Adverse Childhood Experiences and Social Isolation With Later-Life Cognitive Function Among Adults in China

**DOI:** 10.1001/jamanetworkopen.2022.41714

**Published:** 2022-11-11

**Authors:** Li Lin, Bing Cao, Weiqing Chen, Jinghua Li, Yuying Zhang, Vivian Yawei Guo

**Affiliations:** 1 Department of Epidemiology, School of Public Health, Sun Yat-sen University, Guangzhou, China; 2 Department of Neurosurgery, Wu Tsai Neuroscience Institute, Stanford University School of Medicine, Stanford, California; 3 Department of Biostatistics, School of Public Health, Sun Yat-sen University, Guangzhou, China; 4 Department of Child Healthcare, Shenzhen Longhua Maternity and Child Healthcare Hospital, Shenzhen, China

## Abstract

**Importance:**

Studies investigating the association of threat-related and deprivation-related adverse childhood experiences (ACEs) with later-life cognitive decline are lacking.

**Objectives:**

To evaluate the independent association of threat-related and deprivation-related ACEs with cognitive decline over time among middle-aged and older Chinese adults and to examine the modifying role of social isolation in such associations.

**Design, Setting, and Participants:**

This prospective cohort study used cognitive data from the China Health and Retirement Longitudinal Study (CHARLS) baseline survey that was administered between June 1, 2011, and March 31, 2012, and the CHARLS follow-up survey administered between July 1 and September 30, 2015. The life history survey with information of ACEs was additionally administered between June 1 and December 31, 2014. Statistical analysis was performed from March 1 to July 31, 2022. The study population consisted of middle-aged and older adults (age range, 45-97 years) with complete data on ACEs and 2 cognitive assessments and without cognitive impairment at baseline.

**Exposures:**

Five threat-related ACEs (ie, physical abuse, household substance abuse, domestic violence, unsafe neighborhood, and bullying) and 5 deprivation-related ACEs (ie, emotional neglect, household mental illness, incarcerated household member, parental separation or divorce, and parental death) before 17 years of age were queried by questionnaires. The cumulative scores of the 2 ACE dimensions were calculated and grouped into 3 categories as 0, 1, and 2 or more in main analyses.

**Main Outcomes and Measures:**

Cognitive function was measured by episodic memory and executive function. Global cognition was further calculated as the total score of these 2 dimensions. The raw scores of each cognitive test were standardized to *z* scores using baseline means and SDs. Linear mixed-effects models were constructed to examine the association between 2 dimensions of ACEs and the rate of annual cognitive decline. The modifying role of baseline social isolation in such associations was assessed with 3-way interaction tests.

**Results:**

Of the 6466 participants included in main analyses, 3301 (51.1%) were men and the mean (SD) age was 57.2 (8.3) years. Compared with no exposures, experience of 1 deprivation-related ACE was associated with faster cognitive decline in global cognition (β = −0.012 [95% CI, −0.022 to −0.002] SD/y) and executive function (β = −0.010 [95% CI, −0.020 to −0.00002] SD/y), whereas individuals with at least 2 childhood deprivations had faster cognitive declines in all cognitive tests (β = −0.035 [95% CI, −0.050 to −0.019] SD/y for global cognition; β = −0.047 [95% CI, −0.068 to −0.025] SD/y for episodic memory; β = −0.019 [95% CI, −0.034 to −0.004] SD/y for executive function). However, such an association was not observed for threat-related ACEs. In addition, baseline social isolation was a significant modifier in the associations between deprivation-related ACEs and cognitive declines in global cognition (β *=* −0.033 [95% CI, −0.061 to −0.005] SD/y; *P* = .02 for 3-way interaction) and executive function (β = −0.032 [95% CI, −0.059 to −0.005] SD/y; *P* = .02 for 3-way interaction).

**Conclusions and Relevance:**

Deprivation-related ACEs, but not threat-related ACEs, were associated with faster decline in later-life cognitive function, whereas social isolation could modify such detrimental impact. These findings highlight the potential benefits of promoting social integration in maintaining later-life cognitive function among individuals who have experienced childhood deprivation.

## Key Points

**Question:**

Are threat-related and deprivation-related adverse childhood experiences (ACEs) independently associated with later-life cognitive decline, and does social isolation modify such associations?

**Findings:**

In this cohort study of 6466 middle-aged and older adults, individuals with experience of childhood deprivations, but not threats, were susceptible to faster declines in later-life cognitive function over time. The association of deprivation-related ACEs with global cognition and executive function was modified by the level of baseline social isolation.

**Meaning:**

These findings suggest that prioritizing preventive strategies for individuals who are exposed to childhood deprivation along with promotion of social integration may help to minimize the risk of cognitive decline.

## Teaser

This cohort study explores the associations of threat-related and deprivation-related adverse childhood experiences with cognitive decline among middle-aged and older Chinese adults using data from the China Health and Retirement Longitudinal Study.

## Introduction

With a rapid increase of the aging population and prolonged life expectancy in China, age-related morbidities have become major public health concerns.^1^ One such concern is the deterioration of cognitive function, which may not only substantially impair individuals’ well-being^2^ but also could impose an overwhelming burden to their caregivers and families as well as the financial and health systems of society.^3^ According to a nationwide survey conducted between 2015 and 2018 in China, approximately 15.5% of the population 60 years or older had mild cognitive impairment, 6.0% had dementia, and 3.9% had Alzheimer disease.^4^ Therefore, identifying modifiable risk factors associated with cognitive decline is of paramount importance to plan public health interventions and promote healthy aging in China.

Previous research has confirmed several risk factors that could impair normal cognitive function, such as advanced age, lower educational level, unhealthy lifestyles, and physical and mental illnesses.^5^ Evidence has also suggested an association between adverse childhood experiences (ACEs) and reduced cognitive function in later life.^6,7,8,9^ Nevertheless, these studies typically conceptualized ACEs using the cumulative risk model, which suggested that all types of ACEs affect cognitive function through the same underlying mechanism.^6,7,8,9^ An alternative approach, the dimensional model of adversity and psychopathology, was further proposed to differentiate threat-related and deprivation-related ACEs, because these 2 dimensions were considered to be associated with distinct trajectories of neurodevelopment.^10^ Threat-related ACEs involve experiences of harm or violence (eg, abuse, domestic and community violence) that were hypothesized to alter brain areas responsible for emotional processing and regulation.^10,11^ In contrast, deprivation-related ACEs refer to the lack of expected environmental inputs or cognitive stimuli (eg, neglect and absence of a parent) that might affect brain networks associated with language development and executive function.^10,11^ Although several previous studies have revealed stronger associations of deprivation-related ACEs with executive function and cognitive control than threat-related ACEs among children^12,13,14^ or the population with HIV,^15^ whether these 2 dimensions of ACEs exert a different association with cognitive decline in later life among the general population has not been well-articulated.

During the past decade, emerging studies^16,17,18,19^ have striven to identify potential effect modifiers that may modify risks of adverse health outcomes originating from exposure to ACEs. If these factors were confirmed and implemented into intervention, the lifelong negative impact of ACEs on cognitive function might be avoided. Current evidence^17,18,20^ has suggested that social resources such as social support and community cohesion might buffer the cascading impact of ACEs on physical and mental health. In turn, limited social connection or social isolation (eg, few social contacts and small social networks) has been reported as a risk factor for cognitive impairment^21^ and is increasingly prevalent among older adults.^22^ Nevertheless, knowledge regarding the modifying role of social isolation in the associations of childhood threats and deprivations with cognitive decline in later life was limited.

The main aim of this cohort study was to explore the associations of threat-related and deprivation-related ACEs with cognitive decline among middle-aged and older Chinese adults, using data from the China Health and Retirement Longitudinal Study (CHARLS). The modifying role of social isolation in the investigated associations was also evaluated.

## Methods

### Study Design and Population

This cohort study used data from the CHARLS baseline survey administered between June 1, 2011, and March 31, 2012, the life history survey administered between June 1 and December 31, 2014, and the follow-up survey administered between July 1 and September 30, 2015. Briefly, the CHARLS is an ongoing national survey aimed at providing comprehensive information of middle-aged and older adults for aging-related research and policy making.^23^ The baseline survey was performed among 17 708 individuals recruited from 450 villages and resident communities in 28 provinces across China using a multistage probability sampling strategy. Participants were followed up for 4 years during the CHARLS 2015 survey. Information on childhood experiences was additionally collected during the 2014 life history survey. Based on inclusion and exclusion criteria, 6466 participants were eligible for main analyses (age range, 45-97 years). The detailed process of participant selection is listed in the eMethods and eFigure 1 in the Supplement. Ethical approval for the CHARLS was obtained from the institutional review board at Peking University. Each respondent who agreed to participate in the survey provided written informed consent. The study followed the Strengthening the Reporting of Observational Studies in Epidemiology (STROBE) reporting guideline.

### Measurement of Cognitive Function

Cognitive function was measured at 2 time points—the CHARLS 2011 baseline and 2015 follow-up surveys—using questionnaires that were adapted from the Telephone Interview for Cognitive Status.^24^ According to the recommendations by the Health and Retirement Study^25^ and CHARLS,^26^ 2 dimensions of cognitive function were captured: episodic memory and executive function. Episodic memory was measured by immediate recall and delayed recall (score range, 0-10 points, with higher scores indicating better function). Executive function was evaluated by orientation, calculation, and visuospatial ability (score range, 0-11 points, with higher scores indicating better function). Global cognition was defined as the total score of these 2 components with a scale ranging from 0 to 21 points, with higher scores indicating better function. Detailed measurements of each cognitive dimension are provided in the eMethods in the Supplement. To facilitate comparison of cognitive declines across different tests, cognitive raw scores were standardized to *z* scores by subtracting the mean and dividing by the SD at baseline. The continuous *z* scores were used throughout this study as a proxy assessment of cognitive function, with positive values indicating better cognitive performance than the mean population and negative values suggesting cognitive ability inferior to the mean level.

### Definition of ACEs

Information on childhood adversities before 17 years of age were collected through face-to-face interviews in the 2014 life history survey. According to previous studies,^16,27,28^ we captured 10 ACE indicators, including 5 threat-related adversities (ie, physical abuse, household substance abuse, domestic violence, unsafe neighborhood, and bullying) and 5 deprivation-related adversities (ie, emotional neglect, household mental illness, incarcerated household member, parental separation or divorce, and parental death). The detailed definitions of individual ACE indicators are listed in eTable 1 in the Supplement. Each ACE indicator was dichotomized and encoded as 0 for absent or 1 for present. The cumulative score of threat-related and deprivation-related ACEs was further generated by summing the number of ACEs experienced in each dimension. Participants were grouped into 3 categories according to the cumulative scores of the 2 ACE dimensions (ie, 0, 1, and ≥2 threat-related ACEs and 0, 1, and ≥2 deprivation-related ACEs).

### Definition of Social Isolation

An index of social isolation was created based on social networks and social activity or engagement at the 2011 baseline survey.^29^ In line with previous literature, the index includes 4 indicators: living alone, currently unmarried, coming in contact with children less than once a week, and participating in social activities less than once a month.^21,30^ The detailed definitions of each indicator are provided in the eMethods in the Supplement. A total score of social isolation ranging from 0 to 4 was formed by summing these indicators, with higher values representing greater level of isolation. Participants were further classified into 2 categories as nonisolated (score <2) and isolated (score ≥2).^31^

### Covariates

To minimize bias in estimating the association between ACEs and cognitive decline, directed acyclic graphs were applied to select the appropriate adjustment set of covariates using DAGitty software, version 3.0 (developed by Johannes Textor).^32,33^ The final minimally sufficient adjustment set consisted of demographic characteristics (ie, age, sex [recorded by the interviewer], and ethnicity [collected by participant via self-report]) and childhood socioeconomic status (ie, childhood area of residence and parental educational level). Ethnicity was categorized as either Han or ethnic minority group because Han is the most populous ethnic group in China. Childhood area of residence was ascertained by participants’ first household registration status and divided into rural and urban areas. Parental educational level was defined as the highest educational attainment of either parent for each participant and further categorized into illiterate and literate groups.

### Statistical Analysis

Data were analyzed from March 1 to July 31, 2022. Details on descriptive statistics are provided in the eMethods in the Supplement. Changes in cognitive function over time were calculated by subtracting the cognitive *z* scores at follow-up (2015) from those at baseline (2011), which were graphically depicted by different childhood threat and deprivation categories and compared using analysis of variance. In addition, polynomial comparisons were performed to analyze trends in cognitive changes with the number of 2 ACE dimensions increased.

In main analyses, we evaluated the associations of threat-related and deprivation-related ACEs with cognitive decline over time using linear mixed-effects models with complete-case approach. An interaction term of ACEs × follow-up time (year) was added in the models to assess the association between ACEs and the rate of annual cognitive decline (SD per year), whereas random individual-specific intercepts were included to account for within-individual correlations between 2 repeated measurements of cognitive function. Crude models were first constructed, which included the dimensional-specific ACEs, follow-up time, an interaction term of ACEs × follow-up time, and the random intercepts. The adjusted models were further controlled for baseline age, sex, ethnicity, childhood area of residence, and parental educational level. To assess the independent association of childhood threats and deprivations with cognitive decline, the 2 dimensions were also mutually adjusted in the models. Results were presented as β coefficients and 95% CIs.

When a significant association was observed, the secondary analyses were further conducted to evaluate the modifying role of baseline social isolation in the associations between dimensional-specific ACEs and cognitive decline using 3-way interaction tests, with adjustment for the aforementioned covariates. The details of the 3-way interaction test are described in the eMethods in the Supplement. Stratified analyses were further performed by baseline social isolation status if the 3-way interaction test result was statistically significant at *P* < .05.

To adequately address missing data, we generated 60 imputed data sets with the multiple imputation method by chained equations and reanalyzed such associations, following the recommendation to create as many imputations as the percentage of incomplete cases.^34^ All analyses were performed using Stata/SE, version 15.0 (StataCorp LLC). A 2-tailed *P* < .05 was considered statistically significant.

## Results

Of the 6466 participants included in main analyses, 3301 (51.1%) were men and 3165 (48.9%) were women; the mean (SD) age was 57.2 (8.3) years at baseline. Although the magnitude of the differences was relatively small, individuals lost to follow-up or with missing values were older and were more likely to be women, to live in rural areas during childhood, and to have a lower parental educational level than the included participants (eTable 2 in the Supplement). As presented in Table 1, 3300 participants (51.0%) had experienced 1 or more threat-related ACE and 1349 (20.9%) had exposure to at least 2 childhood threats. Compared with no exposures, individuals with experience of 2 or more threat-related ACEs were more likely to be men (813 of 1349 [60.3%] vs 1448 of 3166 [45.7%]), whereas differences in other baseline characteristics between these groups were nonsignificant. The changes of *z* scores in global cognition, episodic memory, and executive function during 4 years were also comparable across the 3 threat-related ACE categories (Figure, A).

**Table 1.** Characteristics of Participants in the China Health and Retirement Longitudinal Study 2011 Survey by Number of Threat-Related and Deprivation-Related ACEs^a^

Characteristics	No. of threat-related ACEs			No. of deprivation-related ACEs		
	0 (n = 3166)	1 (n = 1951)	≥2 (n = 1349)	0 (n = 3219)	1 (n = 2415)	≥2 (n = 832)
Age at baseline, mean (SD), y	57.6 (8.3)	57.0 (8.2)	56.8 (8.2)	56.3 (8.0)	57.7 (8.3)	59.6 (8.5)
Sex						
Men	1448 (45.7)	1040 (53.3)	813 (60.3)	1644 (51.1)	1239 (51.3)	418 (50.2)
Women	1718 (54.3)	911 (46.7)	536 (39.7)	1575 (48.9)	1176 (48.7)	414 (49.8)
Ethnicity						
Han	2928 (92.7)	1803 (92.5)	1263 (93.8)	2948 (91.7)	2271 (94.3)	775 (93.3)
Ethnic minority group	231 (7.3)	146 (7.5)	84 (6.2)	268 (8.3)	137 (5.7)	56 (6.7)
Childhood area of residence						
Rural	2807 (89.9)	1767 (91.5)	1213 (91.3)	2877 (90.5)	2163 (91.0)	747 (90.7)
Urban	314 (10.1)	164 (8.5)	116 (8.7)	302 (9.5)	215 (9.0)	77 (9.3)
Parental educational level						
Illiterate	2656 (89.6)	1658 (90.2)	1133 (89.9)	2713 (88.9)	2053 (90.2)	681 (92.9)
Literate	309 (10.4)	180 (9.8)	127 (10.1)	340 (11.1)	224 (9.8)	52 (7.1)
Baseline cognitive *z* scores, mean (SD)						
Global cognition	0.29 (0.75)	0.28 (0.75)	0.26 (0.73)	0.34 (0.73)	0.27 (0.74)	0.05 (0.76)
Episodic memory	0.20 (0.92)	0.19 (0.91)	0.15 (0.87)	0.24 (0.90)	0.17 (0.91)	0.02 (0.90)
Executive function	0.34 (0.72)	0.33 (0.72)	0.33 (0.71)	0.39 (0.70)	0.33 (0.72)	0.13 (0.75)

Abbreviation: ACE, adverse childhood experience.

^a^ Unless indicated otherwise, data are expressed as No. (%) of participants. Percentages have been rounded and may not total 100, and numbers may not total numbers in column headings owing to missing data.

**Figure.**
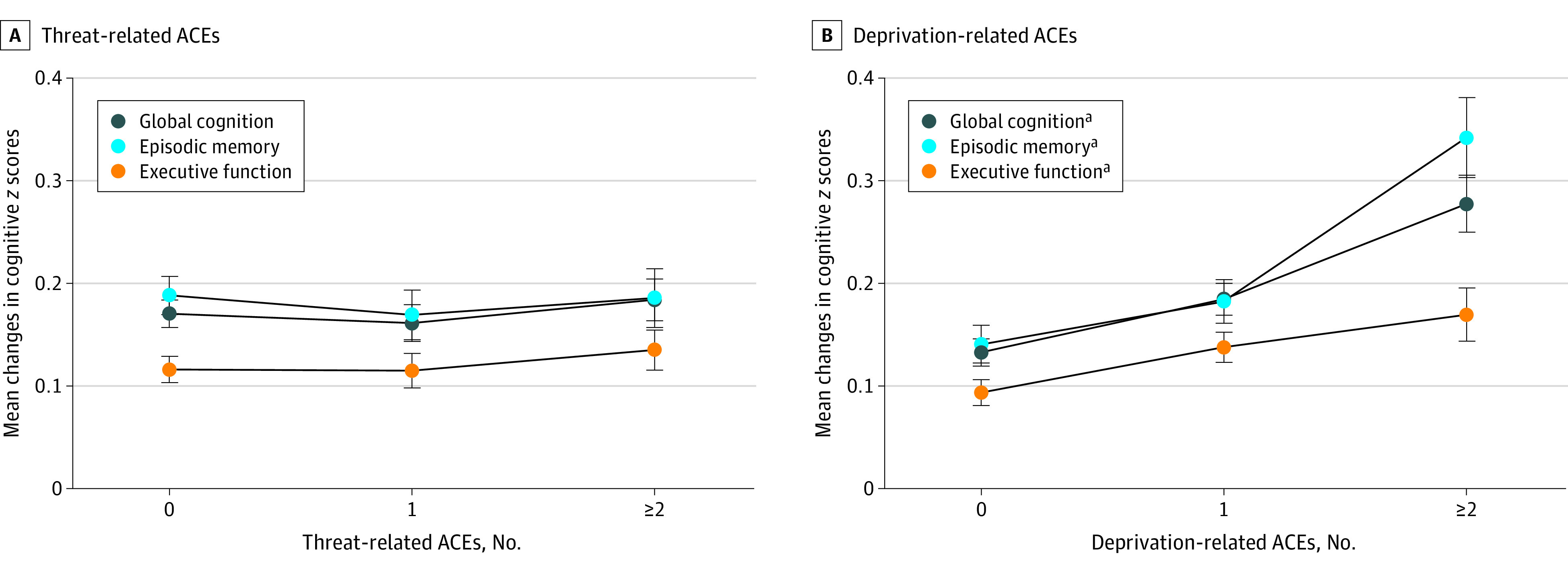
Mean Changes in Cognitive *z* Scores Over Time by Different Groups of Threat-Related and Deprivation-Related Adverse Childhood Experiences (ACEs) Changes in *z* scores of global cognition, episodic memory, and executive function were calculated by subtracting the cognitive *z* scores at follow-up (2015) from those at baseline (2011). Whiskers indicate SEs. ^a^*P* < .05 for difference and trend in cognitive changes across different ACE groups.

In terms of childhood deprivation, 3247 participants (50.2%) were exposed to at least 1 of the 5 deprivation-related ACEs and 832 (12.9%) had experienced 2 or more (Table 1). In general, when compared with the group without any deprivation during childhood, participants with at least 2 types of childhood deprivation were older (mean [SD] age, 59.6 [8.5] vs 56.3 [8.0] years), more likely to have illiterate parents (681 of 733 [92.9%] vs 2713 of 3053 [88.9%]), and more likely to have lower mean (SD) *z* scores of global cognition (0.05 [0.76] vs 0.34 [0.73]), episodic memory (0.02 [0.90] vs 0.24 [0.90]), and executive function (0.13 [0.75] vs 0.39 [0.70]) at baseline. In addition, declines in cognitive *z* scores during the 4-year follow-up tended to be more prominent as the number of deprivation-related ACEs increased; mean changes in cognitive *z* scores from baseline to follow-up increased from 0.13 (0 ACEs) to 0.28 (≥2 ACEs; *P* <.001 for trend) for global cognition, from 0.14 (0 ACEs) to 0.34 (≥2 ACEs; *P* <.001 for trend) for episodic memory, and from 0.09 (0 ACEs) to 0.17 (≥2 ACEs; *P* =.002 for trend) (Figure, B).

Tables 2 and 3 present the associations of threat-related and deprivation-related ACEs with cognitive decline, respectively. Our findings suggest that threat-related ACEs were not associated with global cognition, episodic memory, or executive function at baseline or the rates of annual cognitive declines in any model (Table 2). In contrast, participants with 2 or more deprivation-related ACEs had significantly lower cognitive *z* scores at baseline than those without childhood deprivation in both crude (β = −0.291 [95% CI, −0.353 to −0.228] SD for global cognition; β = −0.216 [95% CI, −0.288 to −0.144] SD for episodic memory; and β = −0.257 [95% CI, −0.316 to −0.198] SD for executive function) and adjusted (β = −0.207 [95% CI, −0.270 to −0.144] SD for global cognition; β = −0.118 [95% CI, −0.192 to −0.044] SD for episodic memory; and β = −0.203 [95% CI, −0.263 to −0.143] SD for executive function) models (Table 3). In addition, compared with no exposure, exposure to 1 deprivation-related ACE was associated with faster cognitive declines in global cognition (β = −0.013 [95% CI, −0.023 to −0.003] SD/y in the crude model; β = −0.012 [95% CI, −0.022 to −0.002] SD/y in the adjusted model) and executive function (β = −0.011 [95% CI, −0.021 to −0.001] SD/y in the crude model; β = −0.010 [95% CI, −0.020 to −0.00002] SD/y in the adjusted model), whereas participants with 2 or more deprivation-related ACEs had faster rates of annual declines in all cognitive dimensions in the crude model (β = −0.036 [95% CI, −0.051 to −0.022] SD/y for global cognition; β = −0.050 [95% CI, −0.070 to −0.030] SD/y for episodic memory; and β = −0.019 [95% CI, −0.033 to −0.005] SD/y for executive function). After adjustment for covariates, the independent associations of 2 or more experiences of childhood deprivation with faster cognitive declines in global cognition (β *=* −0.035 [95% CI, −0.050 to −0.019] SD/y), episodic memory (β *=* −0.047 [95% CI, −0.068 to −0.025] SD/y), and executive function (β *=* −0.019 [95% CI, −0.034 to −0.004] SD/y) remained significant. Therefore, the modifying role of baseline social isolation was only tested in the association between deprivation-related ACEs and cognitive decline.

**Table 2.** Associations Between Threat-Related ACEs and Cognitive Decline Over Time

Cognitive measure	β coefficient (95% CI)^a^	
	Crude model	Adjusted model
Global cognition		
Threat-related ACEs^b^		
0	0 [Reference]	0 [Reference]
1	−0.008 (−0.055 to 0.038)	−0.018 (−0.063 to 0.027)
≥2	−0.030 (−0.083 to 0.023)	−0.042 (−0.093 to 0.010)
Time^c^	−0.043 (−0.049 to −0.036)	−0.041 (−0.048 to −0.034)
Threat-related ACEs × time^c^		
0	0 [Reference]	0 [Reference]
1	0.002 (−0.008 to 0.013)	0.004 (−0.007 to 0.015)
≥2	−0.003 (−0.016 to 0.009)	−0.002 (−0.015 to 0.010)
Episodic memory		
Threat-related ACEs^b^		
0	0 [Reference]	0 [Reference]
1	−0.007 (−0.061 to 0.047)	−0.001 (−0.054 to 0.052)
≥2	−0.048 (−0.109 to 0.013)	−0.023 (−0.084 to 0.038)
Time^c^	−0.047 (−0.056 to −0.038)	−0.044 (−0.054 to −0.035)
Threat-related ACEs × time^c^		
0	0 [Reference]	0 [Reference]
1	0.005 (−0.010 to 0.020)	0.004 (−0.011 to 0.020)
≥2	0.001 (−0.016 to 0.017)	−0.002 (−0.020 to 0.015)
Executive function		
Threat-related ACEs^b^		
0	0 [Reference]	0 [Reference]
1	−0.007 (−0.051 to 0.037)	−0.023 (−0.066 to 0.019)
≥2	−0.012 (−0.061 to 0.038)	−0.041 (−0.090 to 0.008)
Time^c^	−0.029 (−0.035 to −0.023)	−0.029 (−0.036 to −0.022)
Threat-related ACEs × time^c^		
0	0 [Reference]	0 [Reference]
1	0.0001 (−0.010 to 0.011)	0.002 (−0.008 to 0.013)
≥2	−0.005 (−0.016 to 0.007)	−0.001 (−0.013 to 0.011)

Abbreviation: ACE, adverse childhood experience.

^a^ Adjusted models were controlled for baseline age, sex, ethnicity, childhood area of residence, parental educational level, and deprivation-related ACEs. A total of 6466 participants were included in crude models, and 5979 participants with complete data on covariates were included in adjusted models.

^b^ β coefficient and its 95% CI were reported as SD.

^c^ β coefficient and its 95% CI were reported as SD per year.

**Table 3.** Associations Between Deprivation-Related ACEs and Cognitive Decline Over Time

Cognitive measure	β Coefficient (95% CI)^a^	
	Crude model	Adjusted model
Global cognition		
Deprivation-related ACEs^b^		
0	0 [Reference]	0 [Reference]
1	−0.071 (−0.115 to −0.028)	−0.046 (−0.089 to −0.004)
≥2	−0.291 (−0.353 to −0.228)	−0.207 (−0.270 to −0.144)
Time^c^	−0.033 (−0.040 to −0.027)	−0.032 (−0.039 to −0.025)
Deprivation-related ACEs × time^c^		
0	0 [Reference]	0 [Reference]
1	−0.013 (−0.023 to −0.003)	−0.012 (−0.022 to −0.002)
≥2	−0.036 (−0.051 to −0.022)	−0.035 (−0.050 to −0.019)
Episodic memory		
Deprivation-related ACEs^b^		
0	0 [Reference]	0 [Reference]
1	−0.070 (−0.120 to −0.020)	−0.031 (−0.080 to 0.019)
≥2	−0.216 (−0.288 to −0.144)	−0.118 (−0.192 to −0.044)
Time^c^	−0.035 (−0.044 to −0.026)	−0.034 (−0.043 to −0.024)
Deprivation-related ACEs × time^c^		
0	0 [Reference]	0 [Reference]
1	−0.010 (−0.024 to 0.003)	−0.010 (−0.025 to 0.004)
≥2	−0.050 (−0.070 to −0.030)	−0.047 (−0.068 to −0.025)
Executive function		
Deprivation-related ACEs^b^		
0	0 [Reference]	0 [Reference]
1	−0.053 (−0.094 to −0.013)	−0.043 (−0.083 to −0.003)
≥2	−0.257 (−0.316 to −0.198)	−0.203 (−0.263 to −0.143)
Time^c^	−0.023 (−0.030 to −0.017)	−0.023 (−0.029 to −0.016)
Deprivation-related ACEs × time^c^		
0	0 [Reference]	0 [Reference]
1	−0.011 (−0.021 to −0.001)	−0.010 (−0.020 to −0.00002)
≥2	−0.019 (−0.033 to −0.005)	−0.019 (−0.034 to −0.004)

Abbreviation: ACE, adverse childhood experience.

^a^ Adjusted models were controlled for baseline age, sex, ethnicity, childhood area of residence, parental educational level, and threat-related ACEs. A total of 6466 participants were included in crude models, and 5979 participants with complete data on covariates were included in adjusted models.

^b^ β coefficient and its 95% CI were reported as SD.

^c^ β coefficient and its 95% CI were reported as SD per year.

As presented in Table 4, social isolation was shown to be a significant modifier in the association between childhood deprivation and faster rates of annual cognitive decline in global cognition (β *=* −0.033 [95% CI, −0.061 to −0.005] SD/y; *P* = .02 for 3-way interaction) and executive function (β = −0.032 [95% CI, −0.059 to −0.005] SD/y; *P* = .02 for 3-way interaction). However, we did not find the modifying role of social isolation in the association between childhood deprivation and the rate of annual decline in episodic memory (β *=* −0.019 [95% CI, −0.058 to 0.020]; *P* = .34 for 3-way interaction). When stratified by baseline social isolation status, the magnitude of childhood deprivation-related cognitive decline was relatively larger in the socially isolated group than that among participants without social isolation (eFigure 2 in the Supplement). When reanalyzing the dimension-specific associations between ACEs and cognitive decline as well as the modifying role of social isolation with imputed data sets, the results were in line with the main findings (eTables 3-5 in the Supplement).

**Table 4.** Modifying Role of Social Isolation in the Association Between Deprivation-Related ACEs and Cognitive Decline Over Time

Covariate	Cognitive function, β coefficient (95% CI)^a^		
	Global cognition	Episodic memory	Executive function
Deprivation-related ACEs^b^	−0.081 (−0.127 to −0.034)	−0.039 (−0.093 to 0.015)	−0.083 (−0.128 to −0.039)
Social isolation^b^	−0.089 (−0.173 to −0.005)	−0.052 (−0.150 to 0.046)	−0.087 (−0.167 to −0.007)
Time^c^	−0.035 (−0.043 to −0.028)	−0.035 (−0.046 to −0.024)	−0.027 (−0.034 to −0.019)
Deprivation-related ACEs × time^c^	−0.013 (−0.025 to −0.002)	−0.019 (−0.035 to −0.003)	−0.007 (−0.018 to 0.004)
Social isolation × time^c^	0.009 (−0.012 to 0.030)	−0.005 (−0.034 to 0.023)	0.015 (−0.005 to 0.034)
Deprivation-related ACEs × social isolation^b^	−0.036 (−0.150 to 0.078)	0.008 (−0.125 to 0.141)	−0.051 (−0.159 to 0.058)
Deprivation-related ACEs × social isolation × time^c^	−0.033 (−0.061 to −0.005)	−0.019 (−0.058 to 0.020)	−0.032 (−0.059 to −0.005)

Abbreviation: ACE, adverse childhood experience.

^a^ Adjusted models were controlled for baseline age, sex, ethnicity, childhood area of residence, parental educational level, and threat-related ACEs. A total of 5146 participants with complete data on social isolation and covariates were included in adjusted models.

^b^ β coefficient and its 95% CI were reported as SD.

^c^ β coefficient and its 95% CI were reported as SD per year.

## Discussion

The findings of this cohort study suggest that exposure to deprivation-related ACEs was independently associated with a faster rate of annual cognitive decline among middle-aged and older Chinese adults. In contrast, we did not find any association of threat-related ACEs with cognitive decline in later life. These findings further suggest that social isolation could modify the associations of deprivation-related ACEs with declines in global cognition and executive function.

Although the association of ACEs with cognitive function has been consistently reported in different cultural contexts,^6,7,8,9,15^ few studies have investigated the independent role of threat-related and deprivation-related ACEs on later-life cognitive decline in the general population. A cross-sectional study in the US has shown that childhood deprivation, but not threat, was associated with poor cognitive control in adolescents.^13^ Another study of children aged 4 to 7 years^14^ has revealed a deleterious association of deprivation-related adversities on cognitive control, whereas such an association was not significant for exposure to threat. Likewise, evidence from a meta-analysis has demonstrated that early-life deprivation had a stronger association with executive functioning in children compared with their counterparts with experience of threat.^12^ These results are also supported by a case-control study among adults with HIV.^15^ The present study further extends the findings of previous research by demonstrating the long-term association of deprivation-related ACEs, but not threat-related ACEs, with the rate of annual cognitive decline in later life. These findings may provide insight into early identification of the vulnerable group and prioritization of interventions for individuals with experience of childhood deprivation to preserve their cognitive function.

The exact mechanism underlying the different associations of childhood threat and deprivation with cognitive decline in later life remains poorly understood. According to the selective-elimination hypothesis, environmental inputs during childhood could shape neural structure and function by pruning synaptic connections.^35^ When children were raised in a deprived environment characterized by inadequate quantity and complexity of cognitive and social stimuli, some synaptic connections would be infrequently activated and pruned away over time.^36^ Therefore, deprivation-related ACEs could lead to lower numbers of synapses and dysfunctional neural networks, which could further impair the performance of some complex cognition tasks that depend on these areas, such as language ability, working memory, and executive function.^11,37^ Other forms of deprivation (eg, institutional rearing) have also been demonstrated to be associated with decreases in gray matter volume and thickness, especially in areas related to complex cognitive processing, such as prefrontal cortex and anterior cingulate cortex.^38,39^ Therefore, our findings regarding the association between childhood deprivation and cognitive declines in later life were physiologically plausible. In contrast, childhood experience of threat could lead to changes in the neural circuits that are responsible for automatic emotion processing, resulting in maladaptive fear responses and heightened emotional reactivity.^11,37^ Such distinct neurodevelopmental pathways between childhood deprivation and threat provided some explanation for their different associations with cognitive deficits. Nevertheless, more evidence should be provided to elucidate the underlying mechanisms.

Previous studies^17,20^ have revealed that community-level resilience and perceived social support were protective factors for ACE-related problematic behaviors and adverse health outcomes. The present study further extends the findings of existing literature by showing the modifying role of social isolation in the association between childhood deprivation and cognitive decline. Our findings suggest that promoting social integration with larger social networks and more social engagement could alleviate the deleterious outcome of deprivation-related ACEs on faster declines in global cognition and executive function among middle-aged and older adults. The buffering role of social integration could be explained by several possible hypotheses. First, based on the “use it or lose it” theory, participating in social activities was associated with mental stimulation, synaptogenesis, and improved cognitive strategies and skills.^40^ Second, according to the stress-buffering hypothesis, positive social interactions could downregulate the psychological distress originating from ACEs, preserve hippocampal neurons, and reduce neurotoxicity,^41^ which are important factors for maintaining cognitive function with aging.^42^ Third, a low level of social isolation reflects more interpersonal contacts in the social network. Therefore, adults might benefit from social contagion with more healthy practices being adopted, like quitting smoking and drinking, which could subsequently contribute to cognitive preservation.^43,44^ However, we did not observe a significant modifying role of social isolation in the association between deprivation-related ACEs and episodic memory. Existing literature has suggested that episodic memory was more sensitive to age-related cerebral deterioration,^45^ especially in individuals with experience of childhood adversities.^46^ Therefore, the gap in childhood deprivation-induced low episodic memory performance were more likely to increase with aging, regardless of their social isolation status.

### Strengths and Limitations

The strengths of our study include the cohort design, which allowed us to assess the association between ACEs and cognitive decline over time. In addition, we conceptualized ACEs in a dimensional approach, with investigation of differences between distinct types of childhood adversities and later-life cognitive decline.^12,13,14^ Furthermore, we evaluated the modifying role of social isolation, which highlighted the potential benefits of promoting social integration to preserve cognitive function in the presence of childhood deprivation.

Several limitations of this study should be mentioned. First, a large proportion of participants were excluded from main analyses owing to loss of follow-up or missing data, which could introduce selection bias and reduce the generalizability of our study findings. In addition, excluded individuals had experienced relatively higher levels of ACEs and had lower cognitive *z* scores than those included, which may result in an underestimation of the association of childhood threat and deprivation with cognitive decline. Nevertheless, results obtained with imputed data sets were consistent with those of complete-case analyses, indicating the robustness of these findings. Future cohort studies are needed to confirm such associations by improving the study design to reduce missing values and the rates of nonresponse. Second, exposure to ACEs was retrospectively assessed by self-report, which might induce recall bias. However, a previous study^47^ has consistently shown that retrospective measurements of ACEs had good test-retest reliability and may provide some unique and complementary information compared with prospective measures. Third, cognitive function in CHARLS was measured by questions that were partially adapted from the Telephone Interview for Cognitive Status,^24^ rather than a systematic and standardized questionnaire. Therefore, we were only able to use continuous scoring as a proxy assessment of cognitive function. In addition, we included only 2 repeated measurements of cognitive performance of each participant during 4-year follow-up in the linear mixed-effects models, which might reduce the detection power of complex interactions between surveys or growth curve parameters.^48,49^ The association of ACEs with cognitive function, as well as the modifying role of social isolation in such an association, should be further confirmed with clinical diagnosis at multiple time points over a longer period. Fourth, the 95% CIs of the estimated coefficients were broad for the association between childhood deprivation and cognitive decline. It might be explained by the relatively small number of participants with experience of 2 or more deprivation-related ACEs, because the 95% CIs of the estimated coefficients were substantially narrowed in the imputed data sets. Additionally, although we have controlled for several covariates that were selected via directed acyclic graphs in linear mixed-effects models, residual confounding could exist owing to other unmeasured factors that might distort the observed associations.^50^

## Conclusions

The findings of this cohort study suggest that exposure to deprivation-related ACEs, but not threat-related ACEs, is independently associated with faster cognitive decline over time among middle-aged and older Chinese adults. In addition, social isolation may be a significant modifier in the association of childhood deprivation with the rates of annual declines in global cognition and executive function. Our findings underscore the importance of targeted interventions and/or support that align with type-specific risk profiles of ACEs to promote cognitive health among aging population in China. Moreover, our data suggest that promoting social engagement and interaction might be an effective strategy to ameliorate childhood deprivation-associated risks of cognitive declines. However, future randomized clinical trials are needed to confirm these conclusions.

## Notes

Supplement. **eMethods.** Participant Selection, Measurements and Definitions, and Statistical Analysis **eTable 1.** Questionnaire Items of Each Threat-Related and Deprivation-Related ACE Indicator **eTable 2.** Characteristics of Included and Excluded Individuals in the CHARLS 2011 Survey **eTable 3.** Associations Between Threat-Related ACEs and Cognitive Decline Over Time, With Imputed Data Sets **eTable 4.** Associations Between Deprivation-Related ACEs and Cognitive Decline Over Time, With Imputed Data Sets **eTable 5.** Modifying Role of Social Isolation in the Association Between Deprivation-Related ACEs and Cognitive Decline Over Time, With Imputed Data Sets **eFigure 1.** Study Flowchart of Participant Selection **eFigure 2.** Associations Between Deprivation-Related ACEs and Cognitive Decline Over Time, Stratified by Baseline Social Isolation Status
eReferences

## References

[1] Fang EF, Xie C, Schenkel JA, . A research agenda for ageing in China in the 21st century (2nd edition): focusing on basic and translational research, long-term care, policy and social networks. Ageing Res Rev. 2020;64:101174. doi:10.1016/j.arr.2020.101174 32971255 PMC7505078

[2] Yuan L, Zhang X, Guo N, . Prevalence of cognitive impairment in Chinese older inpatients and its relationship with 1-year adverse health outcomes: a multi-center cohort study. BMC Geriatr. 2021;21(1):595. doi:10.1186/s12877-021-02556-5 34696723 PMC8543818

[3] Tochel C, Smith M, Baldwin H, ; ROADMAP consortium. What outcomes are important to patients with mild cognitive impairment or Alzheimer’s disease, their caregivers, and health-care professionals? a systematic review. Alzheimers Dement (Amst). 2019;11:231-247. doi:10.1016/j.dadm.2018.12.003 30906845 PMC6411507

[4] Jia L, Du Y, Chu L, ; COAST Group. Prevalence, risk factors, and management of dementia and mild cognitive impairment in adults aged 60 years or older in China: a cross-sectional study. Lancet Public Health. 2020;5(12):e661-e671. doi:10.1016/S2468-2667(20)30185-7 33271079

[5] Plassman BL, Williams JW Jr, Burke JR, Holsinger T, Benjamin S. Systematic review: factors associated with risk for and possible prevention of cognitive decline in later life. Ann Intern Med. 2010;153(3):182-193. doi:10.7326/0003-4819-153-3-201008030-00258 20547887

[6] Tani Y, Fujiwara T, Kondo K. Association between adverse childhood experiences and dementia in older Japanese adults. JAMA Netw Open. 2020;3(2):e1920740. doi:10.1001/jamanetworkopen.2019.20740 32031646

[7] Schalinski I, Teicher MH, Carolus AM, Rockstroh B. Defining the impact of childhood adversities on cognitive deficits in psychosis: an exploratory analysis. Schizophr Res. 2018;192:351-356. doi:10.1016/j.schres.2017.05.014 28576548

[8] Yang L, Wang Z. Early-life conditions and cognitive function in middle-and old-aged Chinese adults: a longitudinal study. Int J Environ Res Public Health. 2020;17(10):E3451. doi:10.3390/ijerph17103451 32429157 PMC7277849

[9] Ma J, Yang Y, Wan Y, Shen C, Qiu P. The influence of childhood adversities on mid to late cognitive function: from the perspective of life course. PLoS One. 2021;16(8):e0256297. doi:10.1371/journal.pone.0256297 34398901 PMC8366991

[10] Sheridan MA, McLaughlin KA. Dimensions of early experience and neural development: deprivation and threat. Trends Cogn Sci. 2014;18(11):580-585. doi:10.1016/j.tics.2014.09.001 25305194 PMC4252647

[11] McLaughlin KA, Sheridan MA, Lambert HK. Childhood adversity and neural development: deprivation and threat as distinct dimensions of early experience. Neurosci Biobehav Rev. 2014;47:578-591. doi:10.1016/j.neubiorev.2014.10.012 25454359 PMC4308474

[12] Johnson D, Policelli J, Li M, . Associations of early-life threat and deprivation with executive functioning in childhood and adolescence: a systematic review and meta-analysis. JAMA Pediatr. 2021;175(11):e212511. doi:10.1001/jamapediatrics.2021.2511 34309651 PMC8314173

[13] Lambert HK, King KM, Monahan KC, McLaughlin KA. Differential associations of threat and deprivation with emotion regulation and cognitive control in adolescence. Dev Psychopathol. 2017;29(3):929-940. doi:10.1017/S0954579416000584 27424571 PMC5243929

[14] Machlin L, Miller AB, Snyder J, McLaughlin KA, Sheridan MA. Differential associations of deprivation and threat with cognitive control and fear conditioning in early childhood. Front Behav Neurosci. 2019;13:80. doi:10.3389/fnbeh.2019.00080 31133828 PMC6517554

[15] Clark US, Herrington OD, Hegde RR. Effects of early-life adversities on neuropsychiatric and executive functions in HIV-positive adults. J Int Neuropsychol Soc. Published online February 2, 2022. doi:10.1017/S1355617721001466 35105402 PMC10552908

[16] Lin L, Wang HH, Lu C, Chen W, Guo VY. Adverse childhood experiences and subsequent chronic diseases among middle-aged or older adults in China and associations with demographic and socioeconomic characteristics. JAMA Netw Open. 2021;4(10):e2130143. doi:10.1001/jamanetworkopen.2021.30143 34694390 PMC8546496

[17] Longhi D, Brown M, Fromm Reed S. Community-wide resilience mitigates adverse childhood experiences on adult and youth health, school/work, and problem behaviors. Am Psychol. 2021;76(2):216-229. doi:10.1037/amp0000773 33734790

[18] Jaffee SR, Takizawa R, Arseneault L. Buffering effects of safe, supportive, and nurturing relationships among women with childhood histories of maltreatment. Psychol Med. 2017;47(15):2628-2639. doi:10.1017/S0033291717001027 28803556 PMC6293977

[19] Tani Y, Fujiwara T, Kondo K. Adverse childhood experiences and dementia: interactions with social capital in the Japan Gerontological Evaluation Study Cohort. Am J Prev Med. 2021;61(2):225-234. doi:10.1016/j.amepre.2021.01.045 33985835

[20] Cheong EV, Sinnott C, Dahly D, Kearney PM. Adverse childhood experiences (ACEs) and later-life depression: perceived social support as a potential protective factor. BMJ Open. 2017;7(9):e013228. doi:10.1136/bmjopen-2016-013228 28864684 PMC5588961

[21] Yu B, Steptoe A, Chen Y, Jia X. Social isolation, rather than loneliness, is associated with cognitive decline in older adults: the China Health and Retirement Longitudinal Study. Psychol Med. 2021;51(14):2414-2421. doi:10.1017/S0033291720001014 32338228

[22] Donovan NJ, Blazer D. Social isolation and loneliness in older adults: review and commentary of a National Academies Report. Am J Geriatr Psychiatry. 2020;28(12):1233-1244. doi:10.1016/j.jagp.2020.08.005 32919873 PMC7437541

[23] Zhao Y, Hu Y, Smith JP, Strauss J, Yang G. Cohort profile: the China Health and Retirement Longitudinal Study (CHARLS). Int J Epidemiol. 2014;43(1):61-68. doi:10.1093/ije/dys203 23243115 PMC3937970

[24] Fong TG, Fearing MA, Jones RN, . Telephone Interview for Cognitive Status: creating a crosswalk with the Mini-Mental State Examination. Alzheimers Dement. 2009;5(6):492-497. doi:10.1016/j.jalz.2009.02.007 19647495 PMC2783323

[25] McArdle JJ, Fisher GG, Kadlec KM. Latent variable analyses of age trends of cognition in the Health and Retirement Study, 1992-2004. Psychol Aging. 2007;22(3):525-545. doi:10.1037/0882-7974.22.3.525 17874952

[26] Huang Y, Zhang S, Shen J, . Association of plasma uric acid levels with cognitive function among non-hyperuricemia adults: a prospective study. Clin Nutr. 2022;41(3):645-652. doi:10.1016/j.clnu.2021.12.039 35131717

[27] Felitti VJ, Anda RF, Nordenberg D, . Relationship of childhood abuse and household dysfunction to many of the leading causes of death in adults: the Adverse Childhood Experiences (ACE) Study. Am J Prev Med. 1998;14(4):245-258. doi:10.1016/S0749-3797(98)00017-8 9635069

[28] Wolf S, Suntheimer NM. A dimensional risk approach to assessing early adversity in a national sample. J Appl Dev Psychol. 2019;62:270-281. doi:10.1016/j.appdev.2019.03.004

[29] Nicholson NR Jr. Social isolation in older adults: an evolutionary concept analysis. J Adv Nurs. 2009;65(6):1342-1352. doi:10.1111/j.1365-2648.2008.04959.x 19291185

[30] Luo F, Guo L, Thapa A, Yu B. Social isolation and depression onset among middle-aged and older adults in China: moderating effects of education and gender differences. J Affect Disord. 2021;283:71-76. doi:10.1016/j.jad.2021.01.022 33524661

[31] Shen C, Rolls ET, Cheng W, . Associations of social isolation and loneliness with later dementia. Neurology. 2022;99(2):e164-e175. doi:10.1212/WNL.0000000000200583 35676089

[32] Lipsky AM, Greenland S. Causal directed acyclic graphs. JAMA. 2022;327(11):1083-1084. doi:10.1001/jama.2022.1816 35226050

[33] Shrier I, Platt RW. Reducing bias through directed acyclic graphs. BMC Med Res Methodol. 2008;8:70. doi:10.1186/1471-2288-8-70 18973665 PMC2601045

[34] White IR, Royston P, Wood AM. Multiple imputation using chained equations: issues and guidance for practice. Stat Med. 2011;30(4):377-399. doi:10.1002/sim.4067 21225900

[35] Changeux JP, Danchin A. Selective stabilisation of developing synapses as a mechanism for the specification of neuronal networks. Nature. 1976;264(5588):705-712. doi:10.1038/264705a0 189195

[36] Huttenlocher J, Levine S, Vevea J. Environmental input and cognitive growth: a study using time-period comparisons. Child Dev. 1998;69(4):1012-1029. doi:10.1111/j.1467-8624.1998.tb06158.x 9768484

[37] McLaughlin KA, Sheridan MA. Beyond cumulative risk: a dimensional approach to childhood adversity. Curr Dir Psychol Sci. 2016;25(4):239-245. doi:10.1177/0963721416655883 27773969 PMC5070918

[38] McLaughlin KA, Sheridan MA, Winter W, Fox NA, Zeanah CH, Nelson CA. Widespread reductions in cortical thickness following severe early-life deprivation: a neurodevelopmental pathway to attention-deficit/hyperactivity disorder. Biol Psychiatry. 2014;76(8):629-638. doi:10.1016/j.biopsych.2013.08.016 24090797 PMC3969891

[39] Mackes NK, Golm D, Sarkar S, ; ERA Young Adult Follow-up team. Early childhood deprivation is associated with alterations in adult brain structure despite subsequent environmental enrichment. Proc Natl Acad Sci U S A. 2020;117(1):641-649. doi:10.1073/pnas.1911264116 31907309 PMC6955353

[40] Fratiglioni L, Paillard-Borg S, Winblad B. An active and socially integrated lifestyle in late life might protect against dementia. Lancet Neurol. 2004;3(6):343-353. doi:10.1016/S1474-4422(04)00767-7 15157849

[41] Cohen S, Wills TA. Stress, social support, and the buffering hypothesis. Psychol Bull. 1985;98(2):310-357. doi:10.1037/0033-2909.98.2.310 3901065

[42] McEwen BS, Sapolsky RM. Stress and cognitive function. Curr Opin Neurobiol. 1995;5(2):205-216. doi:10.1016/0959-4388(95)80028-X 7620309

[43] Schram MT, Assendelft WJJ, van Tilburg TG, Dukers-Muijrers NHTM. Social networks and type 2 diabetes: a narrative review. Diabetologia. 2021;64(9):1905-1916. doi:10.1007/s00125-021-05496-2 34189591 PMC8241411

[44] Lee Y, Back JH, Kim J, . Systematic review of health behavioral risks and cognitive health in older adults. Int Psychogeriatr. 2010;22(2):174-187. doi:10.1017/S1041610209991189 19883522

[45] Shing YL, Werkle-Bergner M, Brehmer Y, Müller V, Li SC, Lindenberger U. Episodic memory across the lifespan: the contributions of associative and strategic components. Neurosci Biobehav Rev. 2010;34(7):1080-1091. doi:10.1016/j.neubiorev.2009.11.002 19896974

[46] Pan Y. Late-life cognition: do childhood conditions play any role? China Econ Rev. 2020;63:101541. doi:10.1016/j.chieco.2020.101541

[47] Dube SR, Williamson DF, Thompson T, Felitti VJ, Anda RF. Assessing the reliability of retrospective reports of adverse childhood experiences among adult HMO members attending a primary care clinic. Child Abuse Negl. 2004;28(7):729-737. doi:10.1016/j.chiabu.2003.08.009 15261468

[48] Campos-Arias D, De Buyzere ML, Chirinos JA, Rietzschel ER, Segers P. Longitudinal changes of input impedance, pulse wave velocity, and wave reflection in a middle-aged population: the Asklepios Study. Hypertension. 2021;77(4):1154-1165. doi:10.1161/HYPERTENSIONAHA.120.16149 33486987 PMC7946732

[49] Welten M, de Kroon MLA, Renders CM, . Repeatedly measured predictors: a comparison of methods for prediction modeling. Diagn Progn Res. 2018;2:5. doi:10.1186/s41512-018-0024-7 31093555 PMC6460730

[50] Geoffroy M-C, Pinto Pereira S, Li L, Power C. Child neglect and maltreatment and childhood-to-adulthood cognition and mental health in a prospective birth cohort. J Am Acad Child Adolesc Psychiatry. 2016;55(1):33-40.e3. doi:10.1016/j.jaac.2015.10.012 26703907

