# Supplementary material for: Association of Adverse Childhood Experiences and Social Isolation With Later-Life Cognitive Function Among Adults in China
Source: JAMA Netw Open. 2022 Nov 11;5(11):e2241714. doi: 10.1001/jamanetworkopen.2022.41714 (PMC9652754; doi:10.1001/jamanetworkopen.2022.41714)
Supplement: Supplement. — eMethods. Participant Selection, Measurements and Definitions, and Statistical Analysis eTable 1. Questionnaire Items of Each Threat-Related and Deprivation-Related ACE Indicator eTable 2. Characteristics of Included and Excluded Individuals in the CHARLS 2011 Survey eTable 3. Associations Between Threat-Related ACEs and Cognitive Decline Over Time, With Imputed Data Sets eTable 4. Associations Between Deprivation-Related ACEs and Cognitive Decline Over Time, With Imputed Data Sets eTable 5. Modifying Role of Social Isolation in the Association Between Deprivation-Related ACEs and Cognitive Decline Over Time, With Imputed Data Sets eFigure 1. Study Flowchart of Participant Selection eFigure 2. Associations Between Deprivation-Related ACEs and Cognitive Decline Over Time, Stratified by Baseline Social Isolation Status eReferences [file jamanetwopen-e2241714-s001.pdf]

Supplementary Online Content

Lin L, Cao B, Chen W, Li J, Zhang Y, Guo VY. Association of adverse childhood experiences and social isolation with later-life cognitive function among adults in China. *JAMA Netw Open*. 2022;5(11):e2241714. doi:10.1001/jamanetworkopen.2022.41714

eMethods. Participant Selection, Measurements and Definitions, and Statistical Analysis
eTable 1. Questionnaire Items of Each Threat-Related and Deprivation-Related ACE Indicator
eTable 2. Characteristics of Included and Excluded Individuals in the CHARLS 2011 Survey
eTable 3. Associations Between Threat-Related ACEs and Cognitive Decline Over Time, With Imputed Data Sets
eTable 4. Associations Between Deprivation-Related ACEs and Cognitive Decline Over Time, With Imputed Data Sets
eTable 5. Modifying Role of Social Isolation in the Association Between Deprivation-Related ACEs and Cognitive Decline Over Time, With Imputed Data Sets
eFigure 1. Study Flowchart of Participant Selection
eFigure 2. Associations Between Deprivation-Related ACEs and Cognitive Decline Over Time, Stratified by Baseline Social Isolation Status
eReferences

This supplementary material has been provided by the authors to give readers additional information about their work.

© 2022 Lin L et al. *JAMA Network Open*.

eMethods. Participant Selection, Measurements and Definitions, and Statistical Analysis

Participants selection

The baseline survey of CHARLS was administered between June 1, 2011, and March 31, 2012, and the follow-up survey was administered between July 1 and September 30, 2015. Information on adverse childhood experiences (ACEs) was additionally collected in the life history survey, which was administered between June 1 and December 31, 2014.

A total of 17 708 and 20 544 individuals were recruited in the CHARLS 2011 baseline survey and the 2014 life history survey, respectively. Among them, we first conducted 1:1 matching of 14 481 respondents who had completed both surveys. Then, we excluded 396 individuals aged below 45 years or without age information, 2385 individuals with missing values on cognitive function, and 3574 individuals with missing values on ACEs at baseline. Considering that cognitive dysfunction might precede dementia by decades and might be associated with decreased social engagement as well as recall bias of ACEs, we also excluded 845 individuals with physician-diagnosed memory-related diseases (including Alzheimer's disease, Parkinson's disease, and cerebral atrophy) or with a global cognitive z score at or below the 10th percentile at baseline, leaving 7281 eligible participants in the CHARLS 2011 baseline survey. After further exclusion of 429 individuals lost to follow-up and 386 individuals with missing values on cognitive function in the CHARLS 2015 follow-up survey, 6466 participants were finally included in main analyses (age range, 45 to 97 years).

Measurement of Cognitive Function

Episodic memory was measured using immediate and delayed word recall tests. Participants were asked to repeat 10 Chinese words immediately after the interviewer has read to them (score ranged, 0-10 points, with higher scores indicating better function), and recall the same word list as many as they could with a five-minute delay (score ranged, 0-10 points, with higher scores indicating better function). The final score of episodic memory was aggregated into a single score that ranged from 0 to 10 by averaging the immediate and delayed recall points. Executive function was evaluated by two cognitive tasks and a figure drawing test. To evaluate orientation ability, participants were asked to identify current year, month, date, week, and season (score range, 0-5 points, with higher scores indicating better function). For calculation tests, participants were required to subtract 7 from 100 for five times, and 1 point was assigned for each correct answer of the five calculations (score range, 0-5 points, with higher scores indicating better function). To examine visuospatial ability, participants were asked to draw a previously shown picture, and those who successfully reproduced a similar one received 1 point (score range, 0-1 point, with higher scores indicating better function). The score of executive function was generated by summing scores of aforementioned three tasks, with a range of 0-11. The global cognition was defined as the total score of these two components with a scale ranged from 0 to 21. For each task, the raw scores were standardized to z scores using the means and standard deviations (SD) at baseline, with higher z scores indicating better cognitive function.

Definition of Social Isolation

The index of social isolation includes four dichotomized indicators: living arrangement (1 point for living alone), marital status (1 point for currently unmarried, including separated, divorced, widowed, and never married), contacts with children (1 point for contacting with children in person or by phone/email less than once a week), and social participation (1 point for participating in any social activities less than once a month).[2,3] We have extracted six types of social activities during last month from the CHARLS database, including (1)

interacting with friends; (2) playing Mahjong, chess, or cards, or going to community club; (3) going to a sport, social, or other kind of club; (4) taking part in a community-related organization; (5) doing voluntary or charity work; and (6) attending an educational or training course. A total score of social isolation ranging from 0-4 was further formed by summing these dichotomized indicators, with higher values representing greater level of social isolation.

Statistical Analysis
Baseline characteristics of participants were described across different groups of threat-related and deprivation-related ACEs, respectively. Continuous variables were reported as mean ± SD, while categorical variables were described as frequency (percentage). Descriptive statistics of baseline characteristics were further compared between participants included in this study and those excluded due to loss to follow-up or missing values.

When analyzing the modifying role of social isolation, we added three two-way interaction terms (i.e., dimensional-specific ACEs × follow-up time, social isolation × follow-up time, and dimensional-specific ACEs × social isolation) and one three-way interaction term (i.e., dimensional-specific ACEs × social isolation × follow-up time) in linear mixed-effects models simultaneously, with adjustment for baseline age, sex, ethnicity, childhood area of residence, parental educational level, and the other type of ACEs. To facilitate model interpretation for the three-way interaction test and to ensure enough sample in each subgroup, dimensional-specific ACEs were dichotomized into the exposed (i.e., experience of at least one childhood threat or deprivation) and non-exposed group (i.e., without experience of childhood threat or deprivation).

© 2022 Lin L et al. *JAMA Network Open*.

eTable 1. Questionnaire Items of Each Threat-Related and Deprivation-Related ACE Indicator

ACE indicators	Questionnaire Items and definitions
Threat-related ACEs	
Physical abuse	When you were growing up, did your female/male guardian ever hit you? (often[a], sometimes[a], rarely, or never)
Household substance abuse	During the years you were growing up, did your female/male guardian ever have alcoholism or drug? (yes[a] or no)
Domestic violence	Have your father/mother ever beat up your mother/father? (often[a], sometimes[a], not very often, or never)
Unsafe neighborhood	Was it safe being out alone at night in the neighborhood where you lived as a child? (very safe, somewhat safe, not very safe[a], or not safe at all[a])
Bullying	1) When you were a child, how often were you picked on or bullied by kids in your neighborhood? (often[a], sometimes[a], not very often, or never)
	2) When you were a child, how often were you picked on or bullied by kids in your school? (often[a], sometimes[a], not very often, or never)
Deprivation-related ACEs	
Emotional neglect	1) How much love and affection did your female guardian give you while you were growing up? (often, sometimes, rarely[a], or never[a])
	2) How much effort did your female guardian put into watching over you? (a lot, some, a little[a], or not at all[a])
Household mental illness	1) Did your female/male guardian have abnormality of mind when you were young? (yes[a] or no)
	2) During the years you were growing up, had your female/male guardian often showed continued signs of sadness or depression? (during all[a], most[a], some, or only a little of the childhood)
Incarcerated household member	During the years you were growing up, have your female/male guardian ever been arrested or sent to prison? (yes[a] or no)
Parental separation or divorce	Were your biological parents divorced (including long separation due to emotional problems) before you were 17 years? (yes[a] or no)
Parental death[b]	Either of the parents was dead before participant was 17 years. (yes[a] or no)

Abbreviation: ACEs: Adverse Childhood Experiences.

[a]Answers indicate thresholds for ACEs.

[b]Calculated based on dates of birth and their parental death.

© 2022 Lin L et al. *JAMA Network Open*.

eTable 2. Characteristics of Included and Excluded Individuals in the CHARLS 2011 Survey[a]

**Characteristics**	**Included**	**Excluded**[b]
N	6466	10112
Age at baseline, mean (SD), y	57.2 (8.3)	60.3 (10.9)
Missing	0	12
Sex		
Men	3301 (51.1%)	4882 (48.3%)
Women	3165 (48.9%)	5227 (51.7%)
Missing	0	3
Ethnicity		
Han	5994 (92.9%)	7837 (92.5%)
Ethnic minority group	461 (7.1%)	633 (7.5%)
Missing	11	1642
Childhood area of residence		
Rural	5787 (90.7%)	6293 (92.5%)
Urban	594 (9.3%)	513 (7.5%)
Missing	85	3306
Parental educational level		
Illiterate	5447 (89.8%)	5364 (92.7%)
Literate	616 (10.2%)	422 (7.3%)
Missing	403	4326
Social isolation level		
Isolated	4652 (83.5%)	5872 (77.0%)
Non-isolated	921 (16.5%)	1756 (23.0%)
Missing	893	2483
Threat-related ACEs		
0	3166 (49.0%)	1965 (42.6%)
1	1951 (30.2%)	1468 (31.8%)
≥2	1349 (20.9%)	1182 (25.6%)
Missing	0	5496
Deprivation-related ACEs		
0	3219 (49.8%)	1166 (39.1%)
1	2415 (37.3%)	1074 (36.0%)
≥2	832 (12.9%)	741 (24.9%)
Missing	0	7130
Baseline cognitive z scores, mean (SD)		
Global cognition	0.28 (0.74)	-0.07 (1.04)
Episodic memory	0.18 (0.91)	-0.06 (1.04)
Executive function	0.33 (0.72)	-0.11 (1.04)
Missing	0	3407

Abbreviations: ACE, Adverse Childhood Experience; CHARLS, China Health and Retirement Longitudinal Study.

[a]Unless indicated otherwise, data are expressed as No. (%) of participants. Percentages have been rounded and may not total 100.

[b]Excluded individuals were those lost to follow-up or with missing values on the exposure or the outcome.

© 2022 Lin L et al. *JAMA Network Open*.

eTable 3. Associations Between Threat-Related ACEs and Cognitive Decline Over Time, With Imputed Data Sets

**Cognitive measure**	**β Coefficient (95% CI)[a]**	
	**Crude Model**	**Adjusted model**
Global cognition		
Threat-related ACEs[b]		
0	0 [Reference]	0 [Reference]
1	-0.004 (-0.041, 0.033)	-0.022 (-0.056, 0.012)
≥2	-0.035 (-0.076, 0.006)	-0.055 (-0.094, -0.017)
Time[c]	-0.046 (-0.052, -0.041)	-0.047 (-0.052, -0.041)
Threat-related ACEs × Time[c]		
0	0 [Reference]	0 [Reference]
1	-0.003 (-0.012, 0.006)	-0.003 (-0.012, 0.006)
≥2	-0.002 (-0.012, 0.008)	-0.002 (-0.012, 0.008)
Episodic memory		
Threat-related ACEs[b]		
0	0 [Reference]	0 [Reference]
1	-0.005 (-0.050, 0.039)	-0.003 (-0.045, 0.039)
≥2	-0.040 (-0.089, 0.009)	-0.025 (-0.072, 0.021)
Time[c]	-0.056 (-0.063, -0.048)	-0.057 (-0.064, -0.049)
Threat-related ACEs × Time[c]		
0	0 [Reference]	0 [Reference]
1	-0.001 (-0.014, 0.011)	-0.001 (-0.014, 0.011)
≥2	0.000 (-0.014, 0.013)	-0.001 (-0.014, 0.013)
Executive function		
Threat-related ACEs[b]		
0	0 [Reference]	0 [Reference]
1	-0.005 (-0.041, 0.032)	-0.029 (-0.063, 0.005)
≥2	-0.028 (-0.069, 0.012)	-0.063 (-0.102, -0.024)
Time[c]	-0.030 (-0.035, -0.025)	-0.030 (-0.036, -0.025)
Threat-related ACEs × Time[c]		
0	0 [Reference]	0 [Reference]
1	-0.003 (-0.012, 0.006)	-0.003 (-0.012, 0.006)
≥2	-0.003 (-0.013, 0.007)	-0.003 (-0.013, 0.007)

Abbreviation: ACE, Adverse Childhood Experience.

[a]Adjusted models were controlled for baseline age, sex, ethnicity, childhood area of residence, parental educational level, and deprivation-related ACEs.

[b]*β* coefficient and its 95% CI were reported as SD.

[c]*β* coefficient and its 95% CI were reported as SD per year.

© 2022 Lin L et al. *JAMA Network Open*.

eTable 4. Associations Between Deprivation-Related ACEs and Cognitive Decline Over Time, With Imputed Data Sets

**Cognitive measure**	**β Coefficient (95% CI)**[a]	
	**Crude Model**	**Adjusted model**
Global cognition		
Deprivation-related ACEs[b]		
0	0 [Reference]	0 [Reference]
1	-0.115 (-0.150, -0.079)	-0.058 (-0.092, -0.024)
≥2	-0.301 (-0.346, -0.257)	-0.170 (-0.215, -0.125)
Time[c]	-0.038 (-0.044, -0.032)	-0.038 (-0.044, -0.032)
Deprivation-related ACEs × Time[c]		
0	0 [Reference]	0 [Reference]
1	-0.011 (-0.021, -0.002)	-0.012 (-0.022, -0.003)
≥2	-0.034 (-0.045, -0.022)	-0.036 (-0.047, -0.024)
Episodic memory		
Deprivation-related ACEs[b]		
0	0 [Reference]	0 [Reference]
1	-0.098 (-0.138, -0.058)	-0.038 (-0.077, 0.001)
≥2	-0.225 (-0.279, -0.171)	-0.086 (-0.140, -0.033)
Time[c]	-0.044 (-0.052, -0.036)	-0.044 (-0.052, -0.036)
Deprivation-related ACEs × Time[c]		
0	0 [Reference]	0 [Reference]
1	-0.013 (-0.026, 0.000)	-0.014 (-0.026, -0.001)
≥2	-0.047 (-0.063, -0.030)	-0.050 (-0.066, -0.034)
Executive function		
Deprivation-related ACEs[b]		
0	0 [Reference]	0 [Reference]
1	-0.099 (-0.135, -0.064)	-0.056 (-0.091, -0.022)
≥2	-0.283 (-0.329, -0.237)	-0.184 (-0.231, -0.137)
Time[c]	-0.025 (-0.031, -0.019)	-0.025 (-0.030, -0.019)
Deprivation-related ACEs × Time[c]		
0	0 [Reference]	0 [Reference]
1	-0.008 (-0.017, 0.001)	-0.009 (-0.018, 0.0002)
≥2	-0.020 (-0.031, -0.009)	-0.022 (-0.034, -0.010)

Abbreviation: ACE, Adverse Childhood Experience.

[a]Adjusted models were controlled for baseline age, sex, ethnicity, childhood area of residence, parental educational level, and threat-related ACEs.

[b]*β* coefficient and its 95% CI were reported as SD.

[c]*β* coefficient and its 95% CI were reported as SD per year.

© 2022 Lin L et al. *JAMA Network Open*.

eTable 5. Modifying Role of Social Isolation in the Association Between Deprivation-Related ACEs and Cognitive Decline Over Time, With Imputed Data Sets

**Covariate**	**Cognitive function, β coefficient (95% CI)**[a]		
	**Global cognition**	**Episodic memory**	**Executive function**
Deprivation-related ACEs[b]	-0.087 (-0.120, -0.053)	-0.053 (-0.093, -0.013)	-0.088 (-0.122, -0.054)
Social isolation[b]	-0.140 (-0.190, -0.089)	-0.073 (-0.132, -0.015)	-0.146 (-0.196, -0.096)
Time[c]	-0.037 (-0.043, -0.030)	-0.041 (-0.049, -0.033)	-0.025 (-0.031, -0.019)
Deprivation-related ACEs × Time[c]	-0.015 (-0.025, -0.006)	-0.022 (-0.033, -0.010)	-0.010 (-0.019, 0.000)
Social isolation × Time[c]	-0.006 (-0.022, 0.010)	-0.019 (-0.041, 0.003)	0.002 (-0.014, 0.018)
Deprivation-related ACEs × Social isolation[b]	-0.007 (-0.091, 0.078)	-0.014 (-0.109, 0.080)	-0.018 (-0.103, 0.067)
Deprivation-related ACEs × Social isolation × Time[c]	-0.025 (-0.039, -0.011)	-0.013 (-0.042, 0.016)	-0.016 (-0.030, -0.003)

Abbreviation: ACE, Adverse Childhood Experience.

[a]Models were adjusted for baseline age, sex, ethnicity, childhood area of residence, parental educational level, and threat-related ACEs.

[b]*β* coefficient and its 95% CI were reported as SD.

[c]*β* coefficient and its 95% CI were reported as SD per year.

© 2022 Lin L et al. *JAMA Network Open*.

eFigure 1. Study Flowchart of Participant Selection

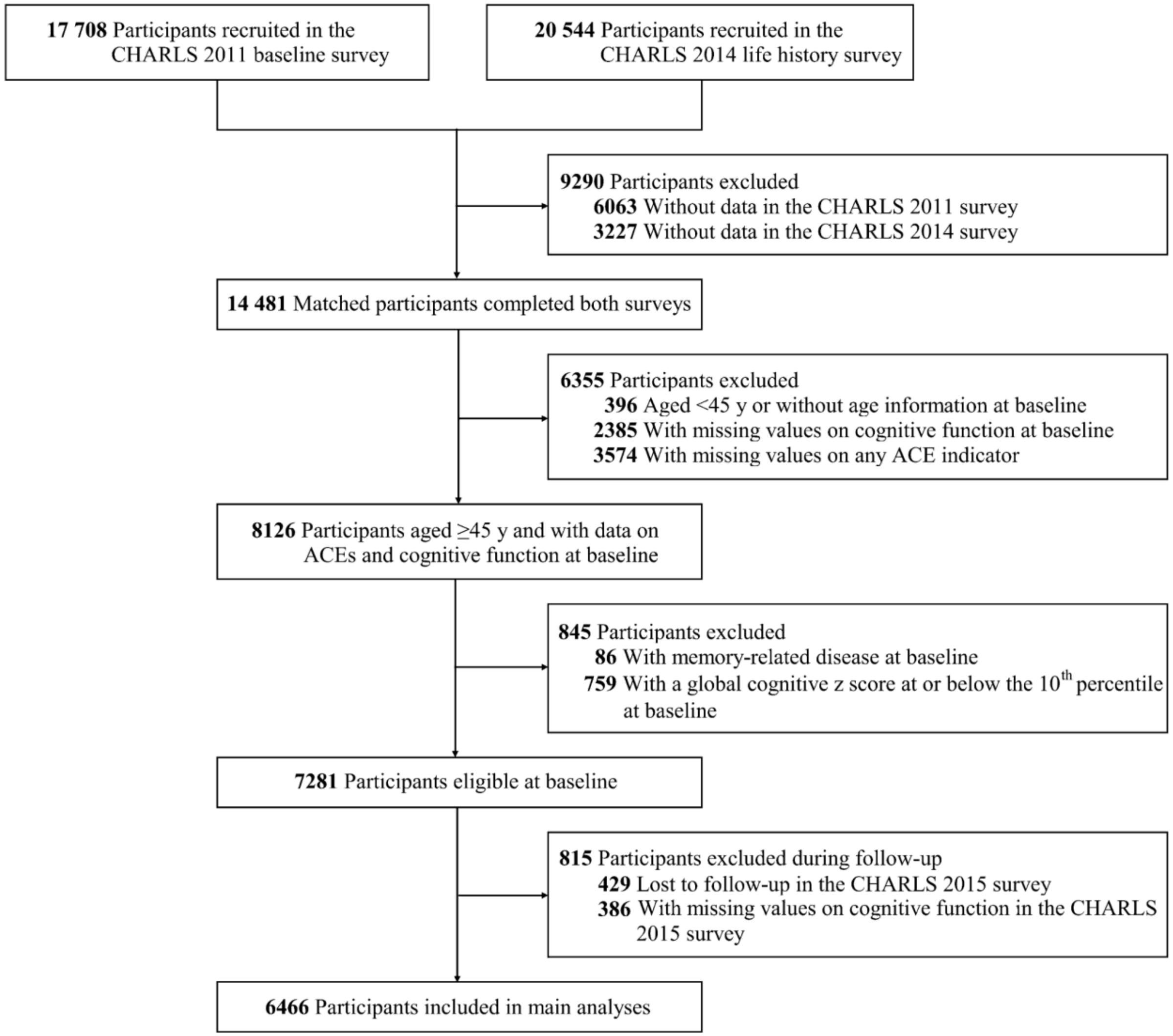

© 2022 Lin L et al. *JAMA Network Open*.

eFigure 2. Associations Between Deprivation-Related ACEs and Cognitive Decline Over Time, Stratified by Baseline Social Isolation Status

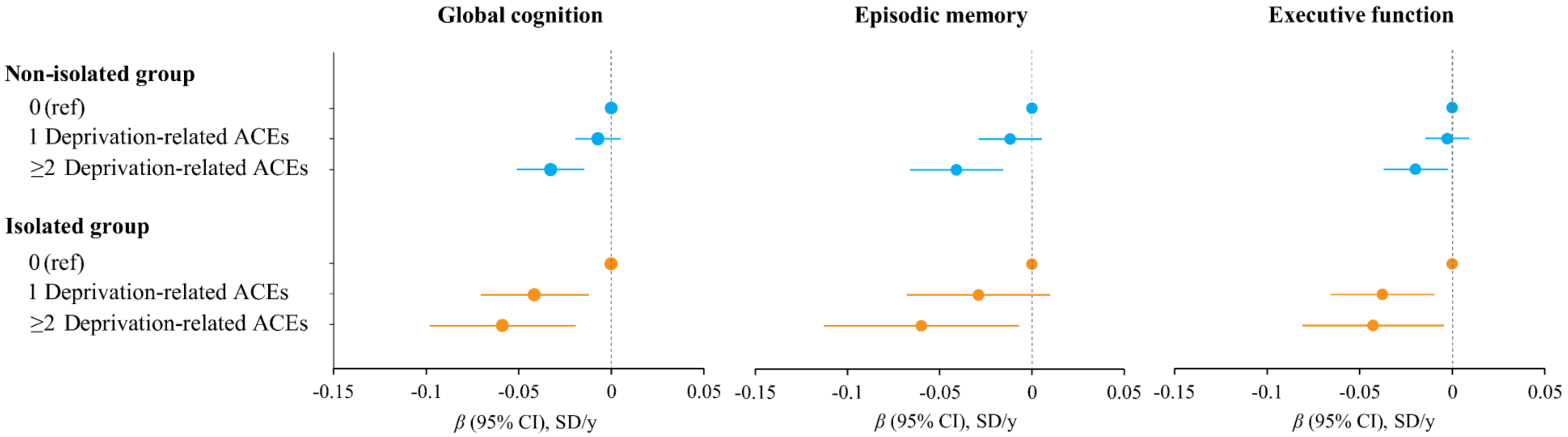

© 2022 Lin L et al. *JAMA Network Open*.

eReferences

1. Zhao Y, Hu Y, Smith JP, Strauss J, Yang G. Cohort profile: the China Health and Retirement Longitudinal Study (CHARLS). *Int J Epidemiol*. Feb 2014;43(1):61-8. doi:10.1093/ije/dys203
2. Yu B, Steptoe A, Chen Y, Jia X. Social isolation, rather than loneliness, is associated with cognitive decline in older adults: the China Health and Retirement Longitudinal Study. *Psychological medicine*. Oct 2021;51(14):2414-2421. doi:10.1017/s0033291720001014
3. Luo F, Guo L, Thapa A, Yu B. Social isolation and depression onset among middle-aged and older adults in China: Moderating effects of education and gender differences. *Journal of affective disorders*. Mar 15 2021;283:71-76. doi:10.1016/j.jad.2021.01.022
4. Shen C, Rolls ET, Cheng W, et al. Associations of Social Isolation and Loneliness With Later Dementia. 2022;99(2):e164-e175. doi:10.1212/WNL.0000000000200583 %J Neurology
5. National Bureau of Statistics of China. Administrative area codes and urban-rural codes for statistics in 2014 (up to October 31, 2014). http://www.stats.gov.cn/tjsj/tjbz/tjyqhdmhcxhfdm/2014/index.html Accessed March 1, 2022.
6. Zhou BF. Predictive values of body mass index and waist circumference for risk factors of certain related diseases in Chinese adults--study on optimal cut-off points of body mass index and waist circumference in Chinese adults. *Biomed Environ Sci*. Mar 2002;15(1):83-96.
7. Andresen EM, Malmgren JA, Carter WB, Patrick DL. Screening for depression in well older adults: evaluation of a short form of the CES-D (Center for Epidemiologic Studies Depression Scale). *Am J Prev Med*. Mar-Apr 1994;10(2):77-84.
8. Chen H, Mui AC. Factorial validity of the Center for Epidemiologic Studies Depression Scale short form in older population in China. *Int Psychogeriatr*. 2014;26(1):49-57. doi:10.1017/S1041610213001701

© 2022 Lin L et al. *JAMA Network Open*.
